# Numerical simulation of bioconvective Darcy Forchhemier nanofluid flow with energy transition over a permeable vertical plate

**DOI:** 10.1038/s41598-022-07254-9

**Published:** 2022-02-25

**Authors:** Ebrahem A. Algehyne, Mounirah Areshi, Anwar Saeed, Muhammad Bilal, Wiyada Kumam, Poom Kumam

**Affiliations:** 1 Department of Mathematics, Faculty of Science, University of Tabuk, P.O. Box 741, Tabuk, 71491 Saudi Arabia; 2 Nanotechnology Research Unit (NRU), University of Tabuk, Tabuk, 71491 Saudi Arabia; 3 Center of Excellence in Theoretical and Computational Science (TaCS-CoE), Faculty of Science, King Mongkut’s University of Technology Thonburi (KMUTT), 126 Pracha Uthit Rd., Bang Mod, Thung Khru, Bangkok, 10140 Thailand; 4 Department of Mathematics, City University of Science and Information Technology, Peshawar, 25000 Pakistan; 5 Applied Mathematics for Science and Engineering Research Unit (AMSERU), Program in Applied Statistics, Department of Mathematics and Computer Science, Faculty of Science and Technology, Rajamangala University of Technology Thanyaburi, Thanyaburi, Pathumthani, 12110 Thailand; 6 Department of Medical Research, China Medical University Hospital, China Medical University, Taichung, 40402 Taiwan

**Keywords:** Engineering, Mathematics and computing

## Abstract

In biological systems, the MHD boundary layer bioconvection flow through permeable surface has several applications, including electronic gadgets, heating systems, building thermal insulation, geological systems, renewable energy, electromagnetism and nuclear waste. The bioconvection caused by the hydromagnetic flow of a special form of water-based nanoliquid including motile microorganisms and nanoparticles across a porous upright moving surface is investigated in this report. The combination of motile microbes and nanoparticles causes nanofluid bioconvection is studied under the cumulative impact of magnetic fields and buoyancy forces. The Brownian motion, thermophoresis effects, heat absorption/generation, chemical reaction and Darcy Forchhemier impact are also unified into the nonlinear model of differential equations. The modeled boundary value problem is numerically computed by employing a suitable similarity operation and the parametric continuation procedure. The parametric study of the flow physical parameters is evaluated versus the velocity, energy, volume fraction of nanoparticles, motile microorganisms’ density, skin friction, Sherwood number and Nusselt number. It has been observed that the velocity profile reduces with the effect of porosity parameter *k*_1_, inertial parameter *k*_2_, Hartmann number and buoyancy ratio. While the energy transition profile significantly enhances with the flourishing values of Eckert number *Ec*, heat absorption/generation *Q* and Hartmann number respectively.

## Introduction

Boundary layer flow across a porous media has numerous implementations in chemical, civil and mechanical engineering, including electronic gadget cooling, heating system, renewable energy, building thermal insulation, geological systems, non-Newtonian biochemical mechanisms, electromagnetism, and underground treatment of waste of atomic or non-nuclear waste, among many others. Thermal transmission augmentation is of current interest in all of these systems from an energy-saving standpoint. Heat transfer may be improved in a variety of methods, including modifying flow shape, boundary conditions, or increasing the fluid's thermal conductivity. Various theoretical and practical research has revealed that suspending greater thermal conductivity micro solid particles improves base fluid heat transfer properties. However, micro-channels erosion and logging arise due to the enormous size of the colloidal materials. As with nanofluids, the use of relatively smaller particulate (nanoparticles) is recommended as a solution to this problem^1–6^. Noghrehabadi et al*.*^7^ addressed the energy transport and entropy production of a nanoliquid over an adiabatic linear shrinking sheet with energy generation. Increases in heat production occur with the upshot of Brownian motion in the region of the sheet, according to the findings. Khan et al*.*^8,9^ reported the rheological effects of Eyring Powell NF as well as the swimming features of gyrotactic microbes on the surface of a porous medium-encased Riga plate. Reza-E-Rabbi et al*.*^10,11^ and Al-Mamun et al.^12^ addressed the hydrodynamic flow characteristics of multiphase radiative Maxwell and Casson fluids across a stretched surface containing nano-sized particles. Bhatti et al*.*^13^ considered the nanoliquid flow with the energy propagating across a Riga plate. The microorganisms and nanofluids saturated in the base fluid are poured into the Riga plate. The results demonstrate that the Rayleigh number and magnetic field of bioconvection diminish the velocity field. Zhang et al*.*^14^ used PCM to investigate the momentum and energy transfer formed by a wavy fluctuation of the surface. Algehyne et al*.*^15^ demonstrated characteristics of MHD Prandtl nanofluid flow on an extended sheet while considering convective boundary conditions. Khan et al*.*^16–18^ demonstrated a 2D flow of bioconvective Eyring–Powell NFs on a upright plate. AlQdah et al.^19^ used the bvp4c method to provide a numerical model of dusty NF flow. To emphasize the influence of physical characteristics on mass and heat transmission, qualitative and quantitative explanations are presented. The mixing effect can greatly boost the thermal conductivity of NFs, according to the propagation data.

The study of fluid flow under the consequences of magnetohydrodynamic (MHD) has a huge implementation in the field of geophysics^20^, earthquakes^21^, astrophysics, sensors, engineering and magnetic drug targeting^22^. Due to its applicability in the biological field, MHD fluid flow in various geometries, rather than industrial applications, relevant to human body parts, is a fascinating and vital scientific subject. Simple flow, pulsatile flow, peristaltic flow, and drug transport are some of the biological applications of MHD^23^. Bilal et al*.*^24^ assessed the convective flow of the hybrid ferrofluid under the principle of electromagnetic induction. Zhou et al*.*^25^ evaluated the MHD Maxwell nanoliquids flow over a porous whirling disc. The energy transmission appears to increase dramatically as the thermophoresis parameter is amplified. Shuaib et al*.*^26^ depicted a 3D nanoliquid communication across two simultaneous spinning plates using hydrodynamics. Their goal was to investigate the cumulative impact of magnetic and electric fields on fluid flow with heat conduction properties. Goyal et al*.*^27^ inspected the issue of threefold diffusive flow having magnetic flux interaction toward a power-law extending sheet using Galerkin finite-element computation. The insights of that study enable industrial corporations in achieving the required product quality by allowing them to manage the frequency of heat transmission. Ghasemi & Hatami^28^ examined the properties of solar radiation on 3D nanoliquid flow across a stretched sheet. The magnetic arena was taken into account, and the nonlinear Rosseland approximation is used to calculate heat radiation. Rasool et al*.*^29^ used the Darcy-Forchheimer correlation to study the temperature communication trends in Jeffery ferrofluid flow across a stretched surface. The electro-magnetic conductivity of the nanoliquid is reinforced by a changing magnetic effect. Small magnetic Reynolds, on the other hand, is thought to negate the generated magnetic effect. Mabood et al*.*^30^ deliberate the characterization of unstable 2D hybrid nanoliquid flow on a smooth strained superficial with the analysis of thermal and MHD effect. The temperature distribution caused by its upward and downstream movement of a flexible spinning disc has been examined by Shuaib et al*.*^31,32^, under the significance of the magnetic field. The MHD hybrid nanofluid flow over different geometries with the bioconvection has been reported by^33–37^.

In biotechnology and biological systems, bioconvection has several applications. The idea of nanofluid bioconvection, which is the subject of the research, illustrates how the simultaneous interplay of denser self-propelled microorganisms, buoyancy forces, and nanoparticles causes spontaneous pattern development and density stratification. Gravitaxis, oxytaxis and gyrotaxis organisms are examples of these microbes. Microscale mixing, Increased mass transfer, especially in micro volumes, and improved NF stability are all advantages of motile microorganisms’ suspension^38^. The oxytactic microorganisms induce hydrodynamic convection, which creates a flow system that transports cells and oxygen from the higher to lower fluid areas. The nanoparticles are not self-driven, and their flow is administered by Brownian motion. As a result, the mobility of motile bacteria appears to be autonomous of the gesture of nanomaterials^39^. The subject of gyrotactic microorganism bioconvection in nanofluids was initially addressed in^39–41^. Kuznetsov^42^ expanded the theory of suspensions by using Buongiorno's conception of bioconvection in nanofluids, which included Brownian motion and thermophoresis. Xu et al*.*^43^ researched an incompressible, stable nanoliquid made up of gyrotactic microorganisms that flowed between parallel surfaces and transferred energy. When the buoyancy convection parameters rise, the velocity profile exhibits a positive reaction. Waqas et al.^44^ & Ramzan et al.^45^ evaluated the ion and Hall slip in a 3D electrically conducting bioconvective NF flow across a stretched sheet under the influence of a magnetic field. Even though the above-mentioned studies have already been concerned with recognizing nanofluid bioconvection, there has been no endeavor in the literature to explore the consequences of Darcy Forchhemier, heat absorption/generation, magnetic field and chemical reaction on nanofluid bioconvection. For biomedical and industrial applications, such a study may provide some visibility into the complicated dynamics of self-propelled microbes in nanoliquid under the presence of external magnetism^46–48^.

The goal of this research is to expand on Olanrewaju and Makinde's work^49–51^ by including hydromagnetic nanofluid and Darcy and heat absorption/generation effect on bioconvection across a vertical permeable plate. Because motile microbes are self-driven, they may swim aggressively in the fluid. Nanoparticles, on the other hand, travel owing to Brownian motion and are transported by the fluid flow. The model is expressed, analyzed, and numerically solved in the parts that follow. The most important findings are graphed and explained.

### Mathematical formulation

**Figure 1:**
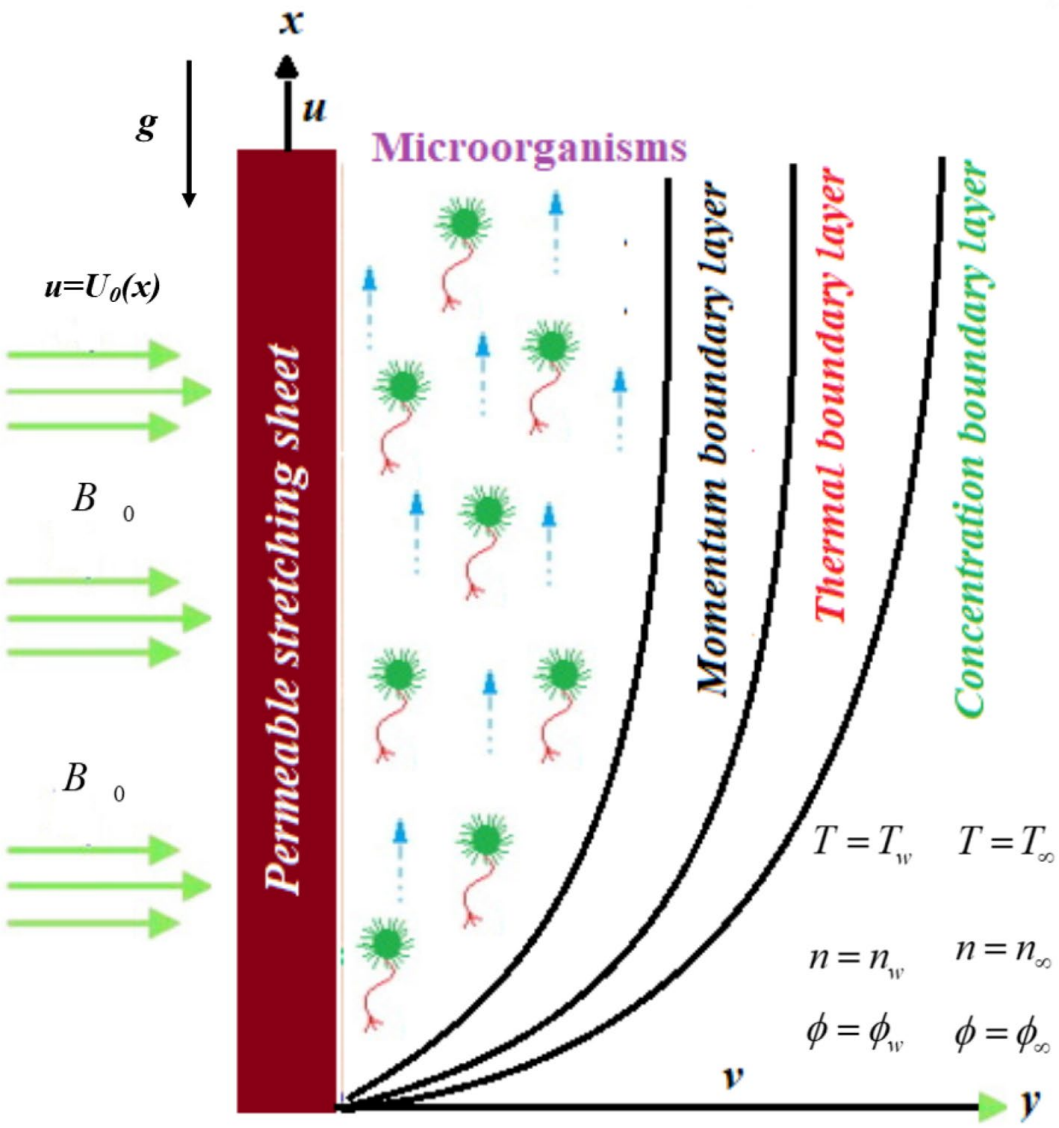
Fluid flow through vertical permeable plate.

The boundary layer gyrostatic microorganisms conducting flow water-based nanoliquid across a vertical permeable plate is addressed in the present work. A constant transversal magnetic field of intensity *B*_0_ is applied to the flow as shown in Fig. 1. The Hall effects and magnetic field are inconsequential since there is no magnetic Reynolds number and voltage is modest. As stated, the presence of nanoparticles is expected to not influence the velocity and direction of microorganisms' movement. The nanoparticulate dispersion is considered to be steady (no nanoparticle coagulation) and dilute (no particulate concentration more than 1%). This is a reasonable postulation because nanoliquid bioconvection is only predicted to occur in a diluted suspension of nanomaterials; otherwise, a high concentration of nanomaterials would raise the base fluid's viscosity, suppressing bioconvection. The framework for bioconvection due to oxytactic microbes is premised on the methodology described in^49–51^. The modeled equations are expressed as:

1 $$ \frac{\partial u}{{\partial x}} + \frac{\partial v}{{\partial y}} = 0, $$

2 $$ \begin{aligned} u\frac{\partial u}{{\partial x}} + v\frac{\partial u}{{\partial y}} & = - \frac{1}{{\rho_{f} }}\frac{\partial p}{{\partial x}} + v_{f} \frac{{\partial^{2} u}}{{\partial y^{2} }} - \frac{{\sigma B_{0}^{2} u}}{{\rho_{f} }} + \frac{1}{{\rho_{f} }}\left[ \begin{gathered} \left( {1 - \phi_{\infty } } \right)\rho_{f} \left( {T - T_{\infty } } \right)\beta g - \left( {\rho_{p} - \rho_{f} } \right) \hfill \\ \left( {\phi - \phi_{\infty } } \right)g - \left( {n - n_{\infty } } \right)\left( {\rho_{m} - \rho_{f} } \right)g\gamma \hfill \\ \end{gathered} \right] \\ & \quad - \frac{\nu }{k}\left( {u - U_{\infty } } \right) - \frac{{k^{\prime}}}{\sqrt k }\left( {u^{2} - U_{\infty }^{2} } \right), \\ \end{aligned} $$

3 $$ u\frac{\partial T}{{\partial x}} + v\frac{\partial T}{{\partial y}} = \alpha \left( {\frac{{\partial^{2} T}}{{\partial x^{2} }} + \frac{{\partial^{2} T}}{{\partial y^{2} }}} \right) + \lambda \left\{ \begin{gathered} D_{B} \frac{\partial \phi }{{\partial y}}\frac{\partial T}{{\partial y}} + \left( {\frac{{D_{T} }}{{T_{\infty } }}} \right) \hfill \\ \left[ {\left( {\frac{\partial T}{{\partial x}}} \right)^{2} + \left( {\frac{\partial T}{{\partial y}}} \right)^{2} } \right] \hfill \\ \end{gathered} \right\} + \frac{\mu \alpha }{k}\left( {\frac{\partial u}{{\partial y}}} \right)^{2} + \frac{{\alpha \sigma B_{0}^{2} u^{2} }}{k} + \frac{{Q_{0} }}{{\left( {\rho c} \right)_{f} }}\left( {T - T_{\infty } } \right), $$

4 $$ u\frac{\partial \phi }{{\partial x}} + v\frac{\partial \phi }{{\partial y}} = D_{B} \left( {\frac{{\partial^{2} \phi }}{{\partial x^{2} }} + \frac{{\partial^{2} \phi }}{{\partial y^{2} }}} \right) + \left( {\frac{{\partial^{2} T}}{{\partial x^{2} }} + \frac{{\partial^{2} T}}{{\partial y^{2} }}} \right)\left( {\frac{{D_{T} }}{{T_{\infty } }}} \right) - k\left( {\phi - \phi_{0} } \right), $$

5 $$ u\frac{\partial n}{{\partial x}} + v\frac{\partial n}{{\partial y}} + \frac{{bW_{c} }}{{\left( {\phi_{w} - \phi_{\infty } } \right)}}\left[ {\frac{\partial }{\partial y}\left( {n\frac{\partial \phi }{{\partial y}}} \right) + \frac{\partial }{\partial x}\left( {n\frac{\partial \phi }{{\partial x}}} \right)} \right] = D_{m} \left( {\frac{{\partial^{2} n}}{{\partial x^{2} }} + \frac{{\partial^{2} n}}{{\partial y^{2} }} + 2\frac{{\partial^{2} n}}{\partial x\partial y}} \right). $$

Here $\left( {u,v} \right)$ determine the velocity component, $\rho_{f}$ is the density, $Q_{0}$ is the heat source, $U_{\infty }$ is the uniform free stream velocity, $\alpha$ is the thermal diffusivity, $k = k_{0} x$ is the Darcy permeability of the permeable medium, $k^{\prime} = \frac{{k_{0} }}{\sqrt x }$ is the Forchhemier resistance,$k_{0}$ is the initial permeability, *D*_*m*_ is the microorganisms diffusivity, *g* and $\beta$ are the gravity and volume expansion, $\sigma$ is the electrical conductivity, $\mu$ is the viscosity, $\lambda = \left( {\rho C} \right)_{p} \left( {\rho C} \right)_{f}$ is the ratio of the heat capacitance to the base fluid, $\gamma$ is the microorganism average volume, *Wc* is cell moving speed, *b* is the chemotaxis coefficient, *n* is the concentration of the microorganisms and $\rho_{m}$ represent their density.

The boundary conditions are:

6 $$ \begin{aligned} & u = U_{0} \left( x \right),\quad v = V,\quad T = T_{w} ,\quad \phi = \phi_{w} ,\quad n = n_{w} \quad {\text{at}}\quad y{ = 0,} \\ & u = 0,\quad v = 0,\quad T \to T_{\infty } ,\quad \phi \to \phi_{\infty } ,\quad n \to n_{w} \quad {\text{as}}\quad y \to \infty . \\ \end{aligned} $$

where $n_{w}$, $\phi_{w}$, $T_{w}$ are the density of motile microbes, volume fraction of nanoparticle and surface temperature. Similarly, the ambient values are signified as $n_{\infty }$, $\phi_{\infty }$, $T_{\infty }$ respectively.

The suction/injection velocity and free stream velocity are presumed as^50^:

7 $$ U_{0} \left( x \right) = a\,x\,\,\,\,\,{\text{and}}\,\,\,\,V = - \left( {a\,v} \right)^{1/2} f_{w} . $$

Here, $a > 0$ is the stretching rate of the plate, $f_{w} = 0$ displays the surface impermeability,$f_{w} > 0$ characterizes suction, and $f_{w} < 0$ represents injection case.

Presenting the subsequent dimensionless variables:

8 $$ \eta = y\left( {a/v} \right)^{1/2} ,\quad \psi = y\left( {av} \right)^{1/2} xf\left( \eta \right),\quad \theta \left( \eta \right) = \frac{{T - T_{\infty } }}{{T_{f} - T_{\infty } }},\quad \zeta \left( \eta \right) = \frac{{\varphi - \varphi_{\infty } }}{{\varphi_{f} - \varphi_{\infty } }},\quad \chi \left( \eta \right) = \frac{{n - n_{\infty } }}{{n_{f} - n_{\infty } }}. $$

We get

9 $$ f^{\prime\prime\prime} + ff^{\prime\prime} - \left( {k_{2} + 1} \right)\left( {f^{\prime}} \right)^{2} + Gr\left( {\theta - \zeta Nr - \chi Rb} \right) - Haf^{\prime} - k_{1} f^{\prime} = 0, $$

10 $$ \theta^{\prime\prime} + \theta^{\prime}\left( {Pr\,f + Nb\zeta^{\prime}} \right) + Nt\left( {\theta^{\prime}} \right)^{2} + Ec\,Pr\,\left( {\left( {f^{\prime\prime}} \right)^{2} + \left( {f^{\prime}} \right)^{2} Ha} \right) + Q\theta = 0, $$

11 $$ \zeta^{\prime\prime} + Le\,f\zeta^{\prime} + \frac{Nt}{{Nb}}\theta^{\prime\prime} - d_{1} \zeta = 0, $$

12 $$ \chi^{\prime\prime} + Lb\,f\chi^{\prime} - Pe\left[ {\zeta^{\prime\prime}\left( {\chi + \Omega } \right) + \zeta^{\prime}\chi^{\prime}} \right] = 0. $$

Here, $\Omega = \frac{{n_{\infty } }}{{n_{w} - n_{\infty } }}$ is the microorganisms concentration difference, $Gr = \frac{{\beta \rho_{f} \left( {1 - \phi_{\infty } } \right)\left( {T_{w} - T_{\infty } } \right)}}{{aU_{0} }}$ is the Grashof number, $Rb = \frac{{\gamma \left( {n_{w} - n_{\infty } } \right)\left( {\rho_{m} - \rho_{f} } \right)}}{{\beta \rho_{f} \left( {1 - \phi_{\infty } } \right)\left( {T_{w} - T_{\infty } } \right)}}$ is the Rayleigh number, $Pe = \frac{{bW_{c} }}{{D_{m} }}$ is the bioconvection Péclet number, $Ec = \frac{{U_{0}^{2} }}{{C_{pf} \left( {T_{f} - T_{\infty } } \right)}}$ is the Eckert number, $Lb = \frac{v}{{D_{m} }}$ is the bioconvection Lewis number, $Le\frac{\nu }{{D_{B} }}$ is the Lewis number, $Pr = \frac{\nu }{\alpha }$ is the Prandtl number, $Ha = \frac{{\sigma B_{0}^{2} }}{{a\rho_{f} }}$ is the Hartmann number, $Nt = \frac{{\lambda D_{T} \left( {T_{w} - T_{\infty } } \right)}}{\alpha }$ is the thermophoresis constraint, $Nb = \frac{{\lambda D_{B} \left( {\varphi_{w} - \varphi_{\infty } } \right)}}{\alpha }$ is the Brownian motion, $Nr = \frac{{\left( {\rho_{f} - \rho_{f\infty } } \right)\left( {\varphi_{w} - \varphi_{\infty } } \right)}}{{\beta \rho_{f} \left( {1 - \varphi_{\infty } } \right)\left( {T_{w} - T_{\infty } } \right)}}$ is the buoyancy ratio,

The transform conditions are:

13 $$ \left. \begin{gathered} f^{\prime}\left( 0 \right) = 1,\quad f\left( 0 \right) = f_{w} ,\quad \zeta \left( 0 \right) = 1,\quad \theta \left( 0 \right) = 1,\quad \chi \left( 0 \right) = 1, \hfill \\ f^{\prime}\left( \infty \right) = 0,\quad \zeta \left( \infty \right) = 0,\quad \theta \left( \infty \right) = 0,\quad \chi \left( \infty \right) = 0. \hfill \\ \end{gathered} \right\} $$

The practical interest quantities of this study are^50^:

14 $$ C_{f} = \frac{{\tau_{w} }}{{\rho_{f} u_{0}^{2} }},\quad Nu = \frac{{xq_{w} }}{{k_{f} \left( {T_{f} - T_{\infty } } \right)}},\quad Sh = \frac{{xq_{m} }}{{D_{B} \left( {\phi_{w} - \phi_{\infty } } \right)}},\quad Nn = \frac{{xq_{n} }}{{\left( {n_{w} - n_{\infty } } \right)D_{n} }}. $$

where $\tau_{w} ,\,\,q_{w} ,\,\,q_{m} ,\,\,q_{n}$ are the skin friction, energy flux, mass flux and motile microorganisms flux defined as:

15 $$ \tau_{w} = \mu \left( {\frac{\partial u}{{\partial y}}|_{y = o} } \right),\,\,q_{w} = - k\left( {\frac{\partial T}{{\partial y}}|_{y = o} } \right),\,\,q_{m} = - D_{B} \left( {\frac{\partial \zeta }{{\partial y}}|_{y = o} } \right),\,\,q_{n} = - Dn\left( {\frac{\partial \chi }{{\partial y}}|_{y = o} } \right). $$

From (14) & (15), we get:

16 $$ \begin{aligned} & C_{fx} = Re_{x}^{1/2} C_{f} = f^{\prime\prime}(0),\quad Nu_{x} = Re_{x}^{ - 1/2} Nu = - \theta^{\prime}(0), \\ & Sh_{x} = Re_{x}^{ - 1/2} Sh = - \zeta^{\prime}(0),\quad Nn_{x} = Re_{x}^{ - 1/2} Nn = - \chi^{\prime}(0). \\ \end{aligned} $$

### Numerical solution

PCM frequently solved the complicated nonlinear boundary value problems that are typically handled by other numerical techniques^52,53^. The subsequent stages demonstrate the essential concept of concerning the PCM approach to a system of ODEs (9–12) & (13).

**Step 1: Reducing the BVP to a first-order system ODEs**

$$ h_{1} = f,\,\,h_{2} = f^{\prime},\,\,h_{3} = f^{\prime\prime},\,\,h_{4} = \theta ,\,\,h_{5} = \theta^{\prime},\,\,h_{6} = \zeta ,\,\,h_{7} = \zeta^{\prime},\,\,h_{8} = \chi ,\,\,h_{9} = \chi^{\prime}. $$

Equations (9–12) are reduced as:

17 $$ \begin{aligned} & h^{\prime}_{3} = - h_{1} h_{3} + \left( {k_{2} + 1} \right)h_{2}^{2} + Hah_{2} - Gr\left( {h_{4} - Nrh_{6} - Rbh_{8} } \right) - k_{1} h_{2} , \\ & h^{\prime}_{5} = - h_{5} \left( {Pr\,h_{1} - Nbh_{7} } \right) - Nth_{5}^{2} - Pr\,Ec\left( {Hah_{2}^{2} } \right) + Qh_{4} , \\ & h^{\prime}_{7} = - Leh_{1} h_{7} d_{1} - \frac{Nt}{{Nb}}\left( { - h_{5} \left( {Pr\,h_{1} + Nbh_{7} } \right) - Nth_{5}^{2} - Pr\,Ec\left( {Hah_{2}^{2} } \right) + Qh_{4} } \right), \\ & h^{\prime}_{9} = - Lbh_{1} h_{9} + Pe\left\{ {\left( {h_{8} + \Omega } \right)\left[ { - Leh_{1} h_{7} - \frac{Nt}{{Nb}}\left[ \begin{gathered} Pr\,h_{1} h_{5} - Nbh_{7} h_{5} - Nth_{5}^{2} \hfill \\ - Pr\,Ec\left( {h_{3}^{2} + Hah_{2}^{2} } \right) + Qh_{4} \hfill \\ \end{gathered} \right]} \right] + h_{7} h_{9} } \right\} \\ \end{aligned} $$

with the boundary conditions.

18 $$ \begin{aligned} & h_{1} \left( 0 \right) = f_{w} ,\quad h_{2} \left( 0 \right) = 1,\quad h_{3} \left( 0 \right) = s_{1} ,\quad h_{4} \left( 0 \right) = 1,\quad h_{5} \left( 0 \right) = s_{2} , \\ & h_{6} \left( 0 \right) = 1,\quad h_{7} \left( 0 \right) = s_{3} ,\quad h_{8} \left( 0 \right) = 1,\quad h_{9} \left( 0 \right) = s_{4} . \\ \end{aligned} $$

**Step 2: Introducing parameter p**

19 $$ \begin{gathered} h^{\prime}_{3} = - h_{1} \left( {h_{3} - 1} \right)p + \left( {k_{2} + 1} \right)h_{2}^{2} + Hah_{2} - Gr\left( {h_{4} - Nrh_{6} - Rbh_{8} } \right) - k_{1} h_{2} , \hfill \\ h^{\prime}_{5} = - \left( {h_{5} - 1} \right)p\left( {Pr\,h_{1} - Nbh_{7} } \right) - Nth_{5}^{2} - Pr\,Ec\left( {Hah_{2}^{2} } \right) + Qh_{4} , \hfill \\ h^{\prime}_{7} = - Leh_{1} \left( {h_{7} - 1} \right)pd_{1} - \frac{Nt}{{Nb}}\left( { - h_{5} \left( {Pr\,h_{1} + Nbh_{7} } \right) - Nth_{5}^{2} - Pr\,Ec\left( {Hah_{2}^{2} } \right) + Qh_{4} } \right), \hfill \\ h^{\prime}_{9} = - Lbh_{1} \left( {h_{9} - 1} \right)p + Pe\left\{ {\left( {h_{8} + \Omega } \right)\left[ { - Leh_{1} h_{7} - \frac{Nt}{{Nb}}\left[ \begin{gathered} Pr\,h_{1} h_{5} - Nbh_{7} h_{5} - Nth_{5}^{2} \hfill \\ - Pr\,Ec\left( {h_{3}^{2} + Hah_{2}^{2} } \right) + Qh_{4} \hfill \\ \end{gathered} \right]} \right] + h_{7} h_{9} } \right\} \hfill \\ \end{gathered} $$

**Step 3: Differentiating by parameter ‘p’**

Furthermore, by employing the implicit scheme, Eq. (19) has been discretized and computed through PCM. The results are shown and discussed in the next section.

## Results and discussion

The discussion segment evaluated the conduct of velocity, energy, nanoparticles concentration and motile microorganism profile as compared to numerous physical. Their out-turns are revealed through the following subsection. While keeping the parameters values are, $k_{1} = 0.1,\,$ $k_{2} = 0.1,\,$ $\Omega = 0.1,\,$ $Gr = 2.0,\,$ $Rb = 0.5,\,$ $Pe = 0.3,\,$ $Ec = 0.1,\,$ $Lb = 0.4,\,$ $Le = 0.1,\,$ $Ha = 1.5,\,$ $Nt =$ $Nb =$ $Nr = 0.3.$

### Velocity profile

**Figure 2:**
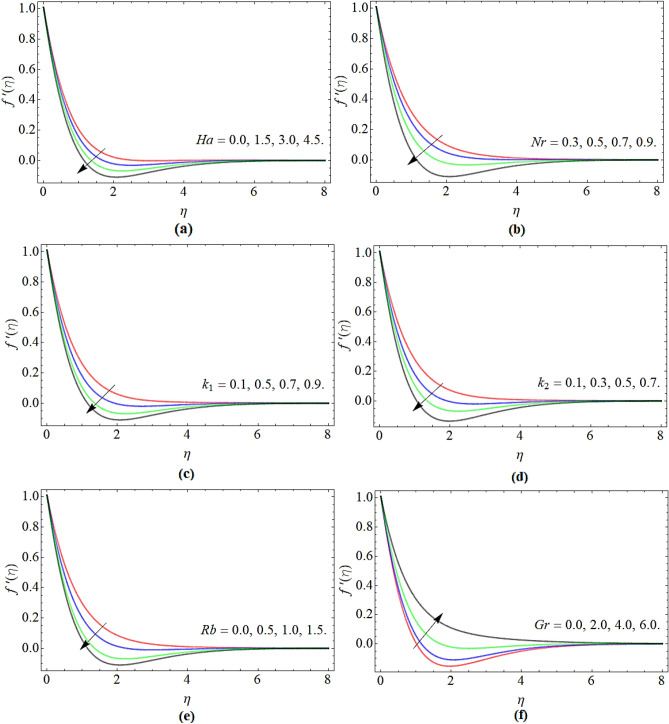
The nature of velocity $f^{\prime}\left( \eta \right)$ profile as opposed to (**a**) Hartmann number *Ha*, (**b**) buoyancy ratio *Nr*, (**c**) porosity parameter *k*_1_, (**d**) inertial parameter *k*_2_, (**e**) bioconvection Rayleigh number *Rb* and (**f**) Grashof number *Gr* respectively.

Figure 2a–f evaluate the nature of velocity $f^{\prime}\left( \eta \right)$ profile versus Hartmann number *Ha,* buoyancy ratio *Nr*, porosity parameter *k*_1_, inertial parameter *k*_2_, bioconvection Rayleigh number *Rb* and Grashof number *Gr* respectively. Figure 2a, b show that the fluid velocity condenses with the effect of Hartmann number and buoyancy ratio. The rising values of Hartmann number *Ha* produce the magnetic effect, where the Lorentz force is fashioned due to magnetic field and that resistive force opposes the fluid flow. The buoyancy effect pushes the fluid towards the sheet edge, reducing both the fluid velocity $f^{\prime}\left( \eta \right)$ and thickness of the momentum boundary layer as publicized in Fig. 2b. Figure 2c, d discovered that the fluid velocity decreases with the upshot of porosity parameter *k*_1_ and inertial constraint *k*_2_. Physically, the porosity effect also produces confrontation to the fluid flow, which results in deacceleration of the velocity field. Figure 2e, f reported that the fluid velocity diminishes with the consequences of bioconvection Rayleigh number and improves with the Grashof number. Both the number have an inverse behavior to each other, that’s why their impact also revealed an opposite trend on the velocity distribution $f^{\prime}\left( \eta \right)$.

### Energy distribution profile

**Figure 3:**
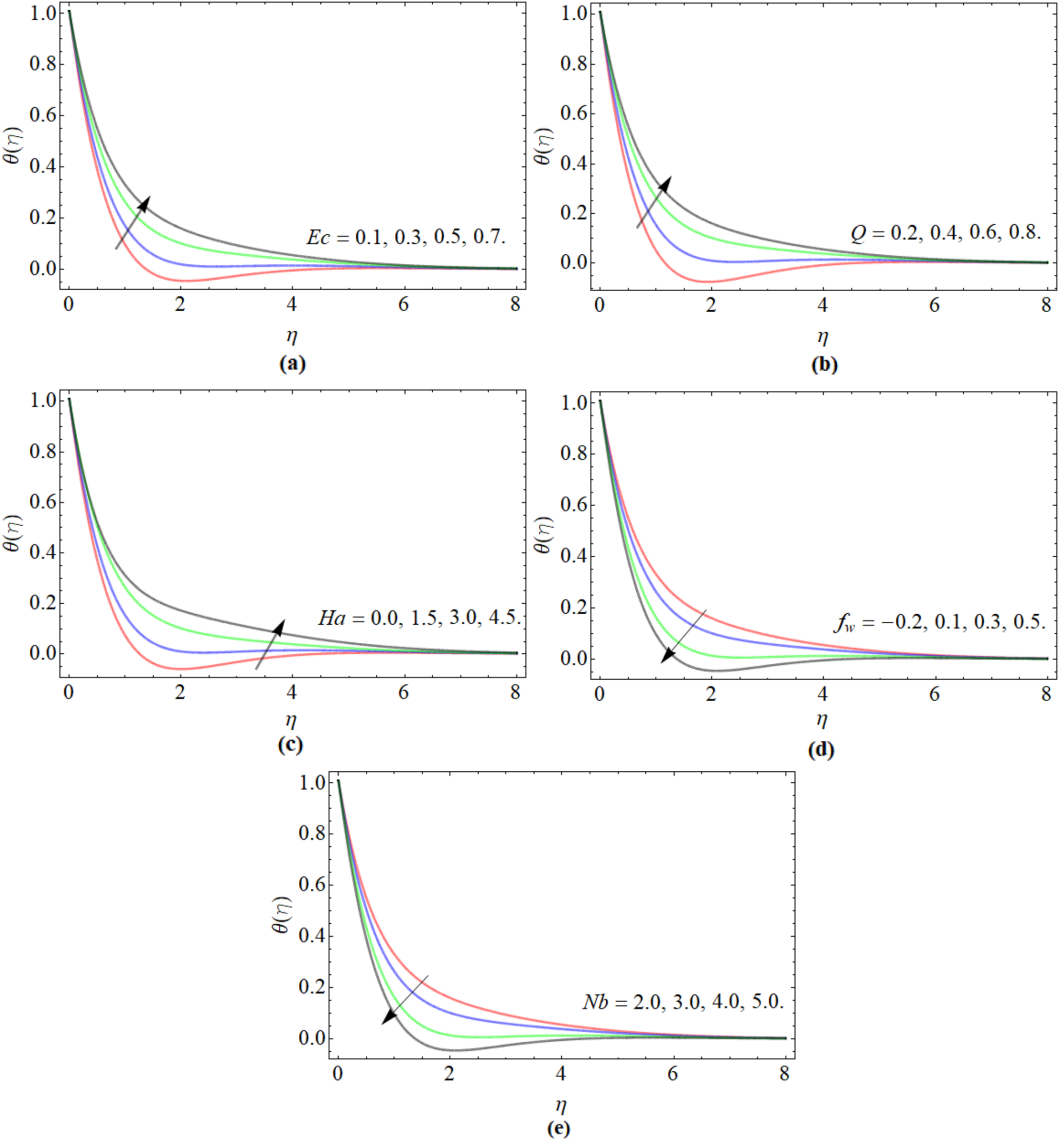
The nature of energy transition $\theta \left( \eta \right)$ profile versus (**a**) Eckert number *Ec*, (**b**) heat absorption/generation *Q*, (**c**) Hartmann number *Ha*, (**d**) suction/injection *f*_*w*_, (**e**) Brownian motion Nb respectively.

Figure 3a–e illustrated the conduct of energy transition $\theta \left( \eta \right)$ profile versus Eckert number *Ec*, heat absorption/generation *Q*, Hartmann number *Ha*, suction/injection *f*_*w*_ and Brownian motion Nb respectively. Figure 3a–c displayed that the energy transition profile significantly enhances with the flourishing values of Eckert number *Ec*, heat absorption/generation *Q* and Hartmann number respectively. The Eckert number reduces the specific heat capacity and heat driving force, which become the reason for temperature enhancement. Similarly, the heat absorption/generation and Hartmann number effect generate extra heat, which causes escalations in the fluid temperature $\theta \left( \eta \right)$. Figure 3d, e manifested that the energy profile diminishes with the rising credit of suction/injection and Brownian motion. During the fluid flow, when suction and injection occurred, the heated particles start evaporating through walls and as a result, the remaining fluid temperature reduces as presented in Fig. 3d.

### Nanoparticles concentration profile

**Figure 4:**
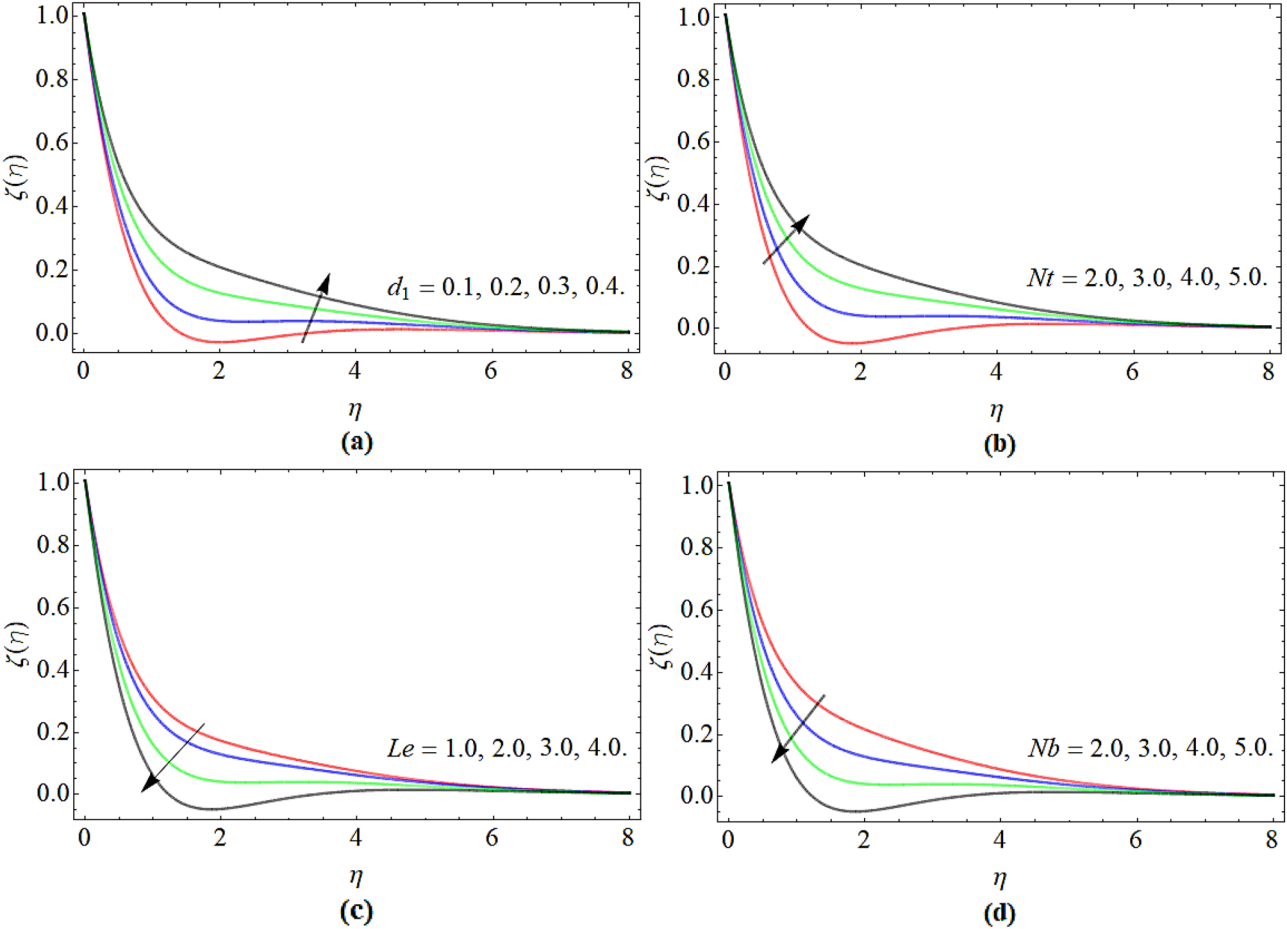
The nature of nanoparticles concentration $\varphi \left( \eta \right)$ profile versus (**a**) chemical reaction *d*_1_, (**b**) thermophoresis constant *Nt*, (**c**) Lewis number *Le*, (**d**) Brownian motion *Nb* respectively.

Figure 4a–d highlighted the nature of nanoparticles concentration $\varphi \left( \eta \right)$ profile versus chemical reaction *d*_1_, thermophoresis constant *Nt*, Lewis number *Le* and Brownian motion *Nb* respectively. Figure 4a, b revealed that the nanoparticles concentration boosts with the effect of chemical reaction *d*_1_ and thermophoresis constant. The variation in chemical reaction accelerates the fluid particles' kinetic energy, which encourages the nanoparticulate profile as spotted in Fig. 4a. On the other hand, the nanoparticles concentration profile significantly decreases with the action of Lewis number. Since the molecular dispersal condenses and kinetic viscosity of fluid upsurges with the Lewis number effect, such a situation has been pragmatic in Fig. 4c, d.

### Motile microorganism profile

**Figure 5:**
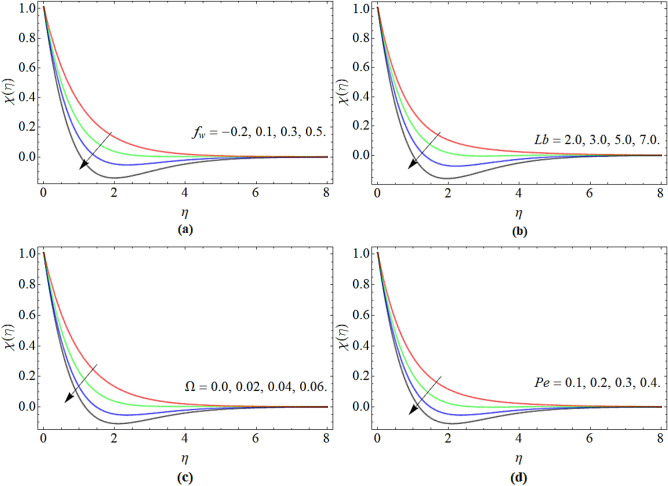
The nature of motile microbe $\chi \left( \eta \right)$ profile versus (**a**) suction/injection *f*_*w*_, (**b**) bio-convection Lewis number *Lb*, (**c**) microbe concentration variance $\Omega$, (**d**) bioconvection Peclet number *Pe* respectively.

Figure 5a–d revealed the nature of motile microorganism $\chi \left( \eta \right)$ profile versus parameters *f*_*w*_, *Lb*, $\Omega$ and *Pe* respectively. Figure 5a–d highlighted that the motile micro-organism profile effectively diminishes with the rising credit of suction/injection, bioconvection Lewis number, microorganism concentration difference and bio-convection Peclet number respectively.

Table 1 displayed the comparative calculations of the current results with the published work.

**Table 1:** Comparative assessment of current outcomes with the published work.

Approximation order	$f^{\prime}\left( 0 \right)$	$- g^{\prime}\left( 0 \right)$
Current result	0.599482	0.600872
Mutuku & Makinde^50^	0.597211	0.609633
Xun et al.^54^	0.510231	0. 615921

## Conclusion

The bio-convection caused by the hydromagnetic flow of a special form of water-based nanoliquid including motile microorganisms and nanoparticles across a porous upright moving surface is investigated. The Brownian motion, thermophoresis effects, heat absorption/generation, chemical reaction and Darcy Forchhemier impact are also unified into the nonlinear model of differential equations. The modeled equations are solved computationally by employing suitable similarity operations and the Parametric continuation Procedure. The following observation has been noticed:

- The velocity profile reduces with the upshot of porosity constraint *k*_1_, inertial parameter *k*_2_, Hartmann number and buoyancy ratio.
- The energy transition profile significantly enhances with the flourishing values of Eckert number *Ec*, heat absorption/generation *Q* and Hartmann number respectively.
- The fluid velocity diminishes with the consequences of bioconvection Rayleigh number and improves with the Grashof number. Both the number has an inverse behavior to each other, that’s why their impact also revealed an opposite trend on the velocity distribution $f^{\prime}\left( \eta \right)$.
- The rising credit of suction/injection, Brownian motion and Prandtl number decrease the energy profile.
- The motile micro-organism profile effectively reduces with the intensifying credit of *f*_*w*_, Lb, $\Omega$, *Pe*.
- The nanoparticles concentration profile boosts with the effect of chemical reaction *d*_1_ and thermophoresis constant, while diminishes with the action of Lewis number *Le* and Brownian motion.

## Data Availability

All data used in this manuscript have been presented within the article.

## List of symbols

*B*_0_: Applied magnetic field
$$\left( {u,v} \right)$$: Velocity component
$Q_{0}$: Heat generation/absorption
$$k = k_{0} x$$: Initial permeability
*g*: Gravity
$$\sigma$$: Electrical conductivity
$$f_{w} < 0$$: Injection
*Wc*: Cell moving speed
*n*: Concentration of the microorganisms
$n_{w}$: Density of motile microbes
$$T_{w}$$: Surface temperature
$$f_{w} = 0$$: Surface impermeability
*Gr*: Grashof number
*Pe*: Péclet number
*Lb*: Bioconvection Lewis number
*Ha*: Hartmann number
*Nb*: Brownian motion
$\lambda$: Ratio of the heat capacitance to the base fluid
$$\rho_{f}$$: Density
*D*_*B*_: Brownian diffusion
$$\alpha$$: Thermal diffusivity
*D*_*m*_: Microorganism’s diffusivity
$$\beta$$: Volume expansion
$$\mu$$: Viscosity
$\gamma$: Microorganism average volume
*b*: Chemotaxis coefficient
$\rho_{m}$: Density of microorganisms.
$\phi_{w}$: Volume fraction of nanoparticle
$$a > 0$$: Stretching rate
$$f_{w} > 0$$: Suction
*Rb*: Rayleigh number
*Ec*: Eckert number
*Le*: Lewis number
*Nt*: Thermophoresis constraint
*Nr*: Buoyancy ratio
$\Omega$: Microorganisms’ concentration difference
